# ASFV Proteins Presented at the Surface of T7 Phages Induce Strong Antibody Responses and Immune Cell Proliferation in *Sus scrofa*

**DOI:** 10.3390/vaccines14010004

**Published:** 2025-12-19

**Authors:** Xinyi Zhang, Yingju Xia, Cheng Wang, Yan Li, Zhifei Li, Lu Xu, Junjie Zhao, Zhen Wang, Xingqi Zou, Xinyv Song, Yuanyuan Zhu, Jinhai Huang, Yebing Liu

**Affiliations:** 1 National/WOAH Reference Laboratory for Classical Swine Fever, China Institute of Veterinary Drug Control, Beijing 100081, China; 3018001380@tju.edu.cn (X.Z.); vet_xiayj@163.com (Y.X.); cheng852365@126.com (C.W.); liyannongda@foxmail.com (Y.L.); lzf1148649754@163.com (Z.L.); xulu777_ivdc@163.com (L.X.); zhaojunjie_ivdc@163.com (J.Z.); wangzhen197711@163.com (Z.W.); zouxingqi@163.com (X.Z.); xiny9226@163.com (X.S.); zhuyuanyzz@163.com (Y.Z.); 2 School of Life Sciences, Tianjin University, Tianjin 300072, China

**Keywords:** African swine fever, vaccine candidate, T7 phage, T cell response

## Abstract

**Background/Objectives:** African swine fever (ASF) causes massive global swine industry losses with no effective vaccine available. This study constructed T7 phages displaying key ASFV proteins to evaluate their potential as an ASF vaccine by assessing viral shedding and immune responses in pigs. **Methods:** Five ASFV proteins were displayed on T7 phages to form VLPs (ASFV-SC-T7 group), with soluble proteins (ASFV-SC group) and PBS as controls; 9 piglets were immunized, boosted at 28 days, challenged with virulent ASFV, and assessed via ELISA, flow cytometry, and real-time PCR. **Results:** ASFV-SC-T7 induced more high-titer antibodies and elevated monocytes/CD8^+^ T cells, but all groups developed ASF lesions, with ASFV-SC-T7 having higher lung/mesenteric lymph node viral loads and no survival improvement (only delayed fever). **Conclusions:** T7 phage-displayed ASFV proteins activate strong immunity, confirming T7 phages as a viable delivery platform, but failed to protect against virulent ASFV, requiring future optimization of antigens and regimens.

## 1. Introduction

African swine fever (ASF), caused by the African swine fever virus (ASFV), is an acute, highly virulent, and lethal infectious disease that poses a severe threat to the global swine industry.

Virulent ASFV strains can cause mortality rates as high as 100% in infected pig populations [1], primarily by inducing hemorrhagic necrosis in key immune organs such as lymph nodes and spleens—with splenic abnormal enlargement and bleeding being hallmark pathological features. As a notifiable disease listed by the World Organization for Animal Health (WOAH) and classified as a Class I animal disease in China, ASF has been dubbed the “top killer of pigs” due to its devastating impact on livestock production systems worldwide.

The global spread of ASF has further amplified its economic toll, with a pivotal turning point occurring in 2018 when the virus emerged in Asia. On August 3 of that year, the News Office of China’s Ministry of Agriculture and Rural Affairs officially announced the first ASF outbreak in Shenyang, Liaoning Province [2]. The virus subsequently spread rapidly across most regions of China, inflicting substantial losses on the country’s pig-farming sector—a trend that mirrors ASF’s broader global impact, from its historical origins in Africa to recent outbreaks in Europe and Southeast Asia [3]. This transcontinental spread underscores the urgent need for effective control strategies, particularly safe and protective vaccines.

ASFV, a member of the family Asfarviridae and genus Asfivirus, is a large nucleocytoplasmic DNA virus and the only known arthropod-borne DNA virus [4]. Its genome consists of a 170–190 kb double-stranded DNA with covalently closed ends, encoding 150–200 viral proteins [1]. The virion exhibits an icosahedral symmetric structure with a complex multilayered architecture, comprising an outer envelope, capsid, inner envelope, nucleocapsid, and nucleoid from exterior to interior [5]. Among the over 200 proteins encoded by ASFV, several play critical roles in viral infection and pathogenesis, while others have been identified as immunogenic antigens—laying the groundwork for vaccine development.

Notably, structural proteins such as P54, P30, and K145R, along with major protective antigens P72 and CD2v, have emerged as key targets for immunogen design. P54, encoded by the E183L gene, contains a transmembrane domain and G-G-X motif; it localizes to the virion inner envelope and viral factories in infected cells, contributing to capsid assembly and regulating programmed cell death. P30 is an early, virulence-associated protein involved in viral internalization and exhibits high immunogenicity. K145R, another structural protein, has been shown in recent studies to be non-essential for ASFV replication in porcine macrophages—yet its deletion delays clinical symptoms and viremia in infected pigs [6,7,8]. P72, a late-stage protein critical for capsid formation, plays a conformation-dependent role in viral entry, and its expression is a marker of active viral replication [9]. CD2v, a glycoprotein and the only known viral surface structural marker, localizes around viral factories during late infection; it interacts with the AP-1 protein complex to modulate viral tissue tropism, immune evasion, and host replication [10]. Collectively, these proteins were selected as immunogens for subsequent immunogenicity assessments due to their roles in viral pathogenesis and immune recognition.

Despite decades of research, current ASFV vaccine strategies remain limited in efficacy and safety. Inactivated vaccines, even when formulated with adjuvants, fail to confer protection against virulent ASFV strains—largely because they cannot elicit robust cellular immune responses [11,12,13]. Subunit vaccines, while precise and safe, face challenges in achieving full protection: although recent studies have identified 44 potential viral polypeptide antigens [14], many lack verified immunogenicity, and even baculovirus-expressed antigens (e.g., CD2v, P54, P30) that induce neutralizing antibodies [15] cannot overcome ASFV’s structural complexity or the multifaceted immune responses required for effective neutralization. DNA vaccines, which elicit both humoral and CD8^+^ T-cell responses, often fail to provide adequate protection despite strong immune activation [16]; prime-boost regimens combining DNA and protein components have also shown insufficient efficacy against virulent ASFV challenges [17,18,19].

Live attenuated vaccines (LAVs)—including naturally attenuated, passaged, and recombinant gene-deletion strains—offer partial promise but carry significant risks. Naturally attenuated strains may cause mild fever, viremia, or chronic infections (especially in immunocompromised animals) and pose risks of abortion and persistent infection [19,20]. Attenuation via serial passage in primary cells (e.g., porcine alveolar macrophages, PAMs) is limited by poor scalability and genetic instability. Recombinant gene-deletion vaccines, while exhibiting promising immunogenicity, show route-dependent efficacy: for example, the ΔDP148R mutant confers protection via intramuscular administration but not intranasal delivery [21]. The only commercially available ASF vaccine, a gene-deletion LAV approved in Vietnam [22], remains controversial due to safety concerns over potential recombination and reversion to virulence. Additionally, LAVs typically provide only homologous protection, with limited cross-protection against heterologous ASFV strains [23].

Against this backdrop, phage display systems have emerged as a promising alternative for vaccine development. Phages are bacterial viruses that can express foreign peptides/proteins on their surfaces, presenting antigens to immune cells in a highly ordered and repetitive manner [24]; they also exhibit immunomodulatory properties, such as reducing reactive oxygen species production and inflammatory cytokine levels [25]. The T7 phage display system, in particular, is widely used in prophylactic and therapeutic vaccine research due to its simplicity, high safety, stability, and ease of storage and transport [26]. T7 phage-expressed peptides/proteins are typically fused to the C-terminus of the capsid protein p10B to minimize steric hindrance, and antigen conjugation via isopeptide bonds enhances stability and stress resistance. Virus-like particles (VLPs) constructed using this system exhibit high antigen purity, robust safety, and potent immunogenicity—capable of eliciting cellular, humoral, and mucosal immune responses [27]. T7 phage-based vaccines have already shown success against pathogens such as Brugia malayi, Zika virus, and foot-and-mouth disease virus, inducing high antibody titers and protective immunity in mouse or rabbit models [27].

Previously, our team constructed five T7 phage-derived VLPs displaying ASFV antigens (P30, P54, P72, CD2v, and K145R) via SpyTag-SpyCatcher-mediated covalent conjugation with the capsid protein P10B [28]. In *BALB/c* mice, these VLPs elicited high titers of antigen-specific IgG, demonstrating strong humoral immunogenicity. However, no studies to date have applied T7 phage display vaccines to pigs—the natural host of ASFV. To address this gap, the present study developed T7 VLPs displaying the same set of ASFV antigens and comprehensively evaluated their immunogenicity in pigs, including assessments of viral shedding (a key marker of transmission risk), humoral immunity (e.g., antigen-specific antibody titers), and cellular immunity (e.g., cytotoxic T lymphocyte (CTL) activity). The primary objective of this research was to determine whether these T7 phage-derived VLPs can confer protection against virulent ASFV challenge, thereby supporting their potential as a novel, safe, and effective ASF vaccine strategy.

## 2. Materials and Methods

### 2.1. Virus and Cell

The challenge virus employed in this study was the 200HAD_50_ HuB/HH/2019 African swine fever virus (ASFV) strain, which was preserved in our laboratory. This strain belongs to the Asfarviridae family and the Asfivirus genus, characterized by its double-stranded DNA genome and high pathogenicity to domestic pigs and wild boars. Phylogenetically, the HuB/HH/2019 strain is classified into genotype II, a lineage that has caused widespread outbreaks in Eurasia since its emergence, with clinical manifestations including high fever (40–42 °C), hemorrhagic lesions in internal organs (e.g., spleen, liver, and lymph nodes), and acute mortality rates exceeding 90% in susceptible pig populations. The 200HAD_50_ titer indicates the virus stock was standardized using the hemadsorption (HAD) assay, where 50% of the tested cell cultures (typically primary porcine alveolar macrophages) showed hemadsorption phenomenon at a dilution corresponding to 200 tissue culture infectious doses, ensuring consistent viral load for challenge experiments. This strain was originally isolated from a clinical sample during an ASF outbreak in Hubei Province, China, in 2019, and has since been maintained through serial passage in susceptible cells under biosafety level 3 (BSL-3) conditions to preserve its biological characteristics, including pathogenicity and immunogenicity, for use in vaccine evaluation, virulence studies, and diagnostic method development.

Porcine alveolar macrophages (PAMs) were isolated as follows: Healthy 4–6-week-old specific pathogen-free piglets were euthanized humanely in accordance with the guidelines of the Institutional Animal Ethics Committee. After sterile thoracotomy to expose the lungs, a sterile catheter was inserted into the trachea, and pre-warmed (37 °C) sterile phosphate-buffered saline (PBS, pH 7.2–7.4) containing 1% penicillin-streptomycin was infused into the alveoli. Lung lobes were gently massaged for 1–2 min, and the bronchoalveolar lavage fluid (BALF) was collected; this lavage process was repeated 3–5 times until the BALF cleared, with 50–100 mL typically obtained per piglet. Pooled BALF was centrifuged at 300× *g* for 10 min at 4 °C to pellet cells, which were then resuspended in RPMI 1640 medium supplemented with 10% fetal bovine serum (FBS) and 1% penicillin-streptomycin. The cell suspension was filtered through a 70 μm cell strainer to remove aggregates and debris, then transferred to sterile culture dishes and incubated in a 5% CO_2_ incubator at 37 °C for 2–4 h to allow PAM adherence. Non-adherent cells were aspirated, and the dish was washed twice with sterile PBS; fresh complete medium was added for continued culture.

### 2.2. Protein Expression and Purification

Plasmids encoding ASFV proteins fused with SpyCatcher (constructed in pET-28a) were preserved in the laboratory [29]. Plasmids were transformed into *E. coli* BL21 (Rosetta) cells, which were cultured in LB medium with ampicillin at 37 °C until OD reached 0.6–0.8. Protein expression was induced with 1 mM isopropyl-β-d-thiogalactopyranoside (IPTG) at 16 °C for 18 h. Bacterial pellets were collected by centrifugation at 4000× *g* for 20 min, resuspended in 30 mL lysis buffer (500 mM NaCl, 50 mM Tris-HCl, 1 mM EDTA, and 1 mM PMSF, pH 8.5), and lysed by sonication (250 W) on ice for 20 min. After centrifugation at 12,000× *g* for 30 min, the supernatants were incubated with nickel-nitrilotriacetic acid (Ni-NTA) columns for binding, followed by elution with gradient imidazole (50 mM Tris-HCl, 200 mM NaCl, 20/60/100/200/500 mM imidazole).

### 2.3. Purification and Construction of ASFV Protein Display on Phage Surface

T7 phages with SpyTag fused to capsid protein P10B (laboratory stock) were inoculated into *E. coli* cultures (OD_600_ = 0.6–0.8) and incubated at 37 °C until visible plaques formed. Cultures were filtered, centrifuged at 8000× *g* for 20 min to collect supernatants, mixed with saturated (NH _3_)_2_SO_4_ (16:9 *v*/*v*), and stirred at 4 °C overnight. After centrifugation at 4000× *g* for 20 min, the pellets were resuspended in 50 mL of PBS to obtain purified T7 phages. Purified T7 phages were mixed with recombinant proteins and incubated overnight at 4 °C to allow SpyTag-SpyCatcher-mediated isopeptide bond formation.

### 2.4. SDS-PAGE and Western Blotting

The samples were mixed with 5 × SDS loading buffer, boiled for 10 min, and centrifuged at 12,000× *g* for 10 min. The supernatants (10 μL) were subjected to SDS-PAGE and Coomassie Brilliant Blue staining. Proteins were transferred to polyvinylidene fluoride (PVDF) membranes at 200 mA for 1 h, washed with 1 × TBST, and blocked with 5% skimmed milk at 37 °C for 1 h. Membranes were incubated overnight at 4 °C with a primary anti-His monoclonal antibody (diluted 1:10,000 in the appropriate blocking buffer). Subsequently, the membranes were washed with Tris-buffered saline containing Tween-20 (TBST) and then incubated for 1 h at 37 °C with a horseradish peroxidase (HRP)-conjugated secondary antibody (diluted 1:20,000 in TBST). After a final wash with TBST, immunoreactive signals were developed using 3,3′,5,5′-tetramethylbenzidine (TMB) substrate and visualized using a gel imaging system.

### 2.5. Animal Experiment and Ethics

Nine 3-week-old piglets tested negative for African swine fever virus (ASFV), classical swine fever virus (CSFV), and porcine reproductive and respiratory syndrome virus (PRRSV), using the real-time PCR method established in our laboratory. All procedures were reviewed and approved by the Animal Welfare and Ethics Committee of the Institute of Veterinary Drug Control (IVDC) (approval number: IVDC(FU) [2023] No. 00219). Experiments involving animals and viruses were performed in the Animal Biosafety Level 3 laboratory (ABSL-3) in the IVDC.

Piglets were randomly divided into three groups (3 pigs/group): the ASFV-SC-T7 group received 2 mL of a mixture of five VLPs (each 5 μg/mL) via intramuscular neck injection in the neck; the ASFV-SC group received 2 mL of a mixture of five ASFV proteins (each 5 μg/mL) via the same route; the control group received 2 mL PBS.

Rectal body temperatures were measured three days prior to immunization, and blood samples were collected from each piglet on the day of immunization. EDTA-treated whole blood and serum samples were collected at 3, 7, 14, and 28 days post-immunization, and peripheral blood mononuclear cells (PBMCs) were isolated from EDTA-treated blood. Booster immunization was administered 28 days after the primary immunization using the same dose and route. Serum and whole blood samples were collected at 3, 7, and 14 days post-booster, with PBMC isolation.

Fourteen days post-booster, the pigs were challenged intramuscularly with 200 hemadsorbing units (HAD_50_) of virulent ASFV. Serum and whole blood samples were collected at 3, 7, and 9 days post-challenge. Oral and rectal swabs and blood samples were processed for viral nucleic acid detection via real-time PCR at 1, 3, 5, 7, 9, 10, 12 days post-challenge. The heart, liver, lung, spleen, kidney, tonsil, submandibular lymph nodes, hilar lymph nodes, gastro-hepatic lymph nodes, mesenteric lymph nodes, and inguinal lymph nodes from euthanized animals were collected for viral load assessment using real-time PCR.

### 2.6. Real-Time Quantitative qPCR

Viral loads in oral/rectal swabs and tissues were quantified by real-time PCR using specific primers and probes based on the B646L gene of ASFV, developed in our laboratory [29]. Briefly, tissue samples or swabs were placed into sterilized tubes containing steel beads and 1 mL of PBS, and then homogenized at 400 strokes per 30 s for three times. The homogenate was then centrifuged at 4000 rpm for seven minutes and 200 μL of the supernatant was collected from each tissue for viral DNA/RNA extraction with Freedom EVO 100 (Tecan, Männedorf, Switzerland) using the DNA/RNA extraction kit (TianGen, Beijing, China). Real-time PCR was conducted using a HyperProbe Mixture (CWBio, Taizhou, China) and specific primers and probes targeting the B646L gene. Quantification of viral copy numbers was derived from RT-qPCR CT values by comparison with a standard curve constructed from a serially diluted pUC-57-B646L gene plasmid.

### 2.7. Enzyme-Linked Immunosorbent Assay (ELISA)

The five proteins, which were purified in our laboratory, were individually diluted in coating buffer and added to 96-well plates at 100 μL per well, followed by coating overnight at 4 °C. After coating, the plates were washed thoroughly with PBST, and non-specific binding sites were blocked with 5% skimmed milk at 37 °C for 1 h. Subsequently, serum samples collected at 14 days post the booster immunization were serially diluted from 1:1000 to 1:512,000 in an appropriate diluent. Each diluted serum sample was added to the wells at 100 μL per well, followed by incubation at 37 °C for 1 h. Following another round of washing with PBST, a 1:10,000-diluted horseradish peroxidase (HRP)-conjugated secondary antibody was added to each well, and the plates were incubated at 37 °C for 1 h (optimal incubation condition for secondary antibody binding). After removing unbound secondary antibody by thorough washing with PBST, TMB substrate solution was added to initiate the colorimetric reaction. The reactions were terminated by the addition of sulfuric acid, and the optical density at 450 nm (OD_450_) was measured using a microplate reader to complete the ELISA.

### 2.8. Flow Cytometric Analysis

PBMCs were stained with 2 μL of monoclonal antibodies against CD3-PerCP (BD, Heidelberg, Germany), CD4-PE (BD, Germany), CD8-FITC (Bio-Rad, Hercules, CA, USA), CD163-FITC (Bio-Rad, USA), CR2-PE (ANTIBODIES, Osaka, Japan), and monocyte-Alexa Fluor 647 (Bio-Rad, USA) at 4 °C in the dark for 30 min, followed by flow cytometric analysis using a BD Accuri™ C6 Plus flow cytometer. The FACS data were analyzed using FlowJo_10.9.0 software.

### 2.9. Biostatistical Analysis

All statistical analyses were performed using SPSS 26.0 software (IBM Corp., Armonk, NY, USA) and GraphPad Prism 10 (GraphPad Software, San Diego, CA, USA). Quantitative data, including ASFV-specific antibody titers and other target immunological indicators, are presented as the mean ± standard deviation (mean ± SD). This study focused on comparing the differences in target indicators between two experimental groups (p30-T7 VLP-immunized group and p54-T7 VLP-immunized group) and the control group (PBS-immunized group), with no direct comparison between the two experimental groups. Independent samples *t*-tests were used for pairwise comparisons between each experimental group and the control group, respectively. Since two independent *t*-tests were conducted (i.e., p30-T7 vs. PBS and p54-T7 vs. PBS), this constituted a multiple comparison scenario. To control the risk of Type I error (false positive) associated with multiple testing, the Bonferroni correction method was applied to adjust the *p*-values, with the corrected significance level set at α = 0.05/2 = 0.025. A corrected *p*-value < 0.025 was considered statistically significant.

## 3. Results

### 3.1. Construction of ASFV Protein Present on T7 Phage Surface

**Figure 1:**
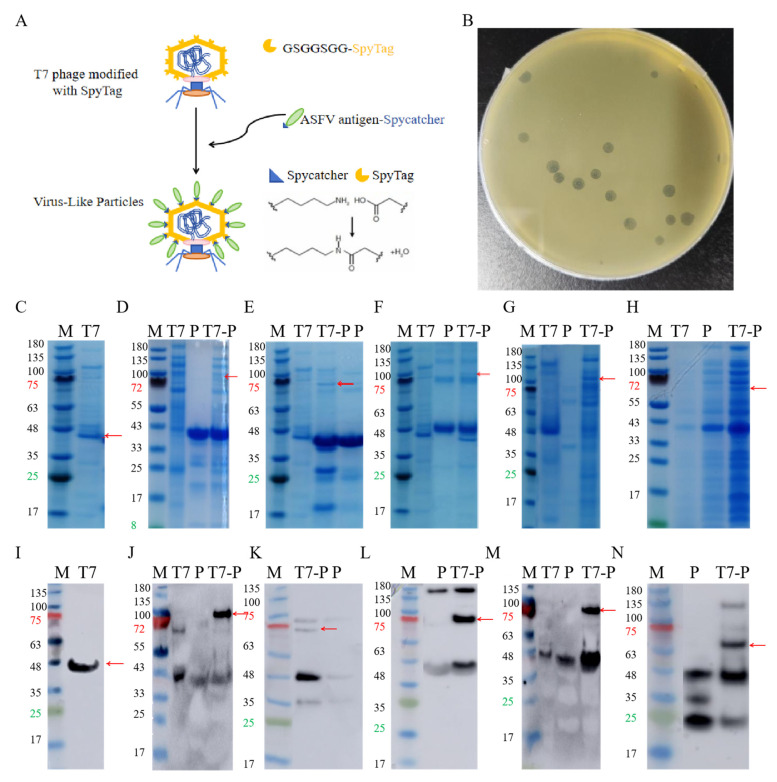
**Purification of antigen proteins and VLP construction. (A**). Construction of T7 phage VLPs. (**B**). Plaque, counting. (**C**). PAGE of T7 phage, The red arrow indicates the P10B band. (**D**). PAGE of P30 and the ligation product, the red arrow indicates the ligation product. (**E**). PAGE of P54 and the ligation product, the red arrow indicates the ligation product. (**F**). PAGE of P72 and the ligation product, the red arrow indicates the ligation product. (**G**). PAGE of CD2v and the ligation product, the red arrow indicates the ligation product. (**H**). PAGE of K145R and the ligation product, the red arrow indicates the ligation product. (**I**). Western Blot of T7 phage, The red arrow indicates the P10B band. (**J**). Western Blot of P30 and the ligation product, the red arrow indicates the ligation product. (**K**). Western Blot of P54 and the ligation product, the red arrow indicates the ligation product. (**L**). Western Blot of P72 and the ligation product, the red arrow indicates the ligation product. (**M**). Western Blot of CD2v and the ligation product , the red arrow indicates the ligation product. (**N**). Western Blot of K145R and the ligation product , the red arrow indicates the ligation product.

ASFV antigens were displayed on T7 phages through SpyTag-SpyCatcher-mediated isopeptide bridging, where SpyTag was fused to the capsid protein P10B, and SpyCatcher was conjugated to the C-terminus of each ASFV antigen [29] (Figure 1A). The expected molecular weight of the P10B-SpyTag fusion protein was 40 kDa, and SDS-PAGE confirmed the successful phage purification (Figure 1C,I). The expected molecular weights of p30, p54, p72, CD2v, and K145R were 37 kDa, 421 kDa, 45 kDa, 35 kDa, and 32 kDa, respectively. Most proteins matched the expected sizes; K145R showed a slightly higher molecular weight but specifically bound to the anti-His antibody (Figure 1D–H,J–N), confirming successful purification. The conjugation of ASFV proteins to T7 phages was verified by SDS-PAGE and Western blotting (red arrows), confirming the construction of five T7 phage-displayed ASFV proteins. The stability and stress resistance of these VLPs have been validated via transmission electron microscopy (TEM) and plaque assays in previous reports [29].

### 3.2. T7 Phage-Displayed ASFV Proteins Elicit Robust Humoral and Cellular Immunity

Experimental pigs were randomly allocated to three groups (*n* = 3 per group). Pigs in the ASFV-SC-T7 group received a 2 mL intramuscular injection in the cervical region containing a mixture of five types of VLPs at a total concentration of 250 μg/mL (50 μg/mL per VLP). Pigs in the ASFV-SC group were administered 2 mL of a mixture of five ASFV proteins at the same total concentration (250 μg/mL) via the same route. Pigs in the control group were injected with 2 mL of PBS.

Baseline body temperature was recorded for three days prior to immunization, and baseline blood samples were collected on day 0. Anticoagulated whole blood and serum were obtained 3, 7, 14, and 28 days after the primary immunization. Peripheral blood mononuclear cells (PBMCs) were isolated from the collected whole blood. A booster immunization was administered on day 28, using the same dose and route as the primary immunization. Serum and whole blood samples were collected at 3, 7, and 14 days after the booster, followed by PBMC isolation.

**Figure 2:**
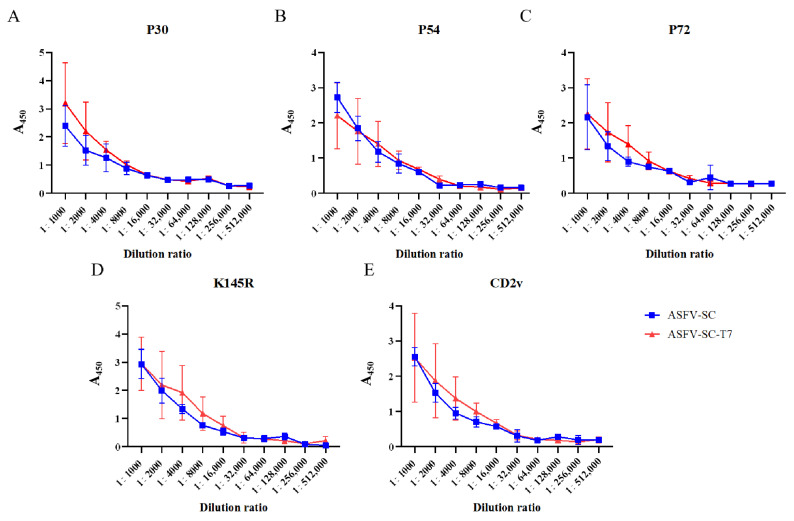
Antibody dynamics in immunized pigs. Antibody titers in the ASFV-SC group and ASFV-SC-T7 group were determined at 14 days post the second immunization. (**A**). With an A450 value >0.3 as the cutoff, the P30 antibody titers in both the ASFV-SC group and ASFV-SC-T7 group were 1:128,000. (**B**). With an A450 value >0.3 as the cutoff, the P54 antibody titer was 1:16,000 in the ASFV-SC group and 1:32,000 in the ASFV-SC-T7 group. (**C**). With an A450 value >0.3 as the cutoff, the P72 antibody titer was 1:64,000 in the ASFV-SC group, while the P54 antibody titer in the ASFV-SC-T7 group was 1:32,000. (**D**). With an A450 value >0.3 as the cutoff, the K145R antibody titers in both the ASFV-SC group and ASFV-SC-T7 group were 1:32,000. (**E**). With an A450 value >0.3 as the cutoff, the CD2v antibody titers in both the ASFV-SC group and ASFV-SC-T7 group were 1:32,000.

ELISA results demonstrated that both individual ASFV proteins and their corresponding VLP formulations elicited high titers of antibodies following booster immunization (Figure 2A–E). Using an absorbance (A450) value > 0.3 as the cutoff, the results of specific antibody titer detection in pigs from the ASFV-SC group and ASFV-SC-T7 group at 14 days post the second immunization are summarized as follows: the P30 antibody titers were consistent in both groups, reaching 1:128,000; the P54 antibody titer was 1:16,000 in the ASFV-SC group, while it was increased to 1:32,000 in the ASFV-SC-T7 group (twice that of the control group); the P72 antibody titer was 1:64,000 in the ASFV-SC group and 1:32,000 in the ASFV-SC-T7 group; and the K145R and CD2v antibody titers were consistent in both groups, being 1:32,000 and 1:32,000, respectively. In conclusion, both the ASFV-SC group and the ASFV-SC-T7 group induced high levels of specific antibodies, indicating a robust humoral immune response (The relevant data are shown in Supplementary Table S1).

**Figure 3:**
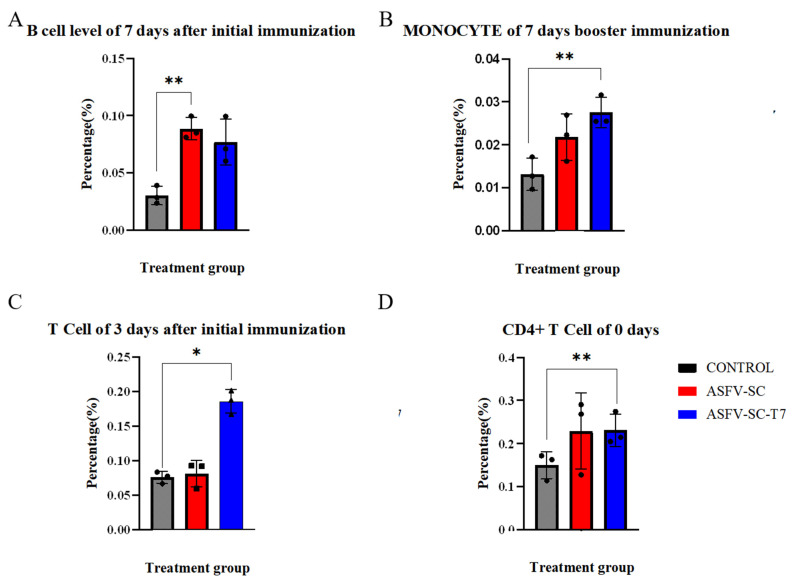
Immune cell profiling of immunized pigs. The abscissa represents the time points after immunization, and the ordinate denotes the percentage of target cells relative to peripheral blood mononuclear cells (PBMCs). (**A**) Proportion of B cells in PBMCs on day 7 following primary immunization. (**B**). Proportion of monocytes in PBMCs on day 7 after booster immunization. (**C**). Proportion of CD8^+^ T cells in PBMCs at the indicated time points after primary and booster immunizations. (**D**). Proportion of CD4^+^ T cells in PBMCs before immunization. * *p* < 0.025, ** *p* < 0.005 (*n* = 3).

On day 7 after the primary immunization, the proportion of B cells in PBMCs was significantly higher (*p* < 0.01) in the ASFV-SC group than in the control group (Figure 3A), with values of (8.8 ± 0.98)% and (3.0 ± 0.79)%, respectively. An independent two-tailed *t*-test indicated a statistically significant difference between groups (t = 21.08, *p* < 0.01). This time point coincided with the initial increase in antibody levels. Given that B cells develop in the bone marrow and migrate to peripheral lymphoid organs upon maturation, the elevated proportion of B cells in PBMCs suggests cell amplification and active migration induced by the ASFV-SC subunit vaccine.

Monocyte frequency is a well-established indicator of innate immune activation [28]. Seven days post-booster, the ASFV-SC-T7 group showed a significantly higher proportion of monocytes in PBMCs compared to the control group (Figure 3B): (2.8 ± 0.35)% versus (1.3 ± 0.38)%. Statistical analysis confirmed a significant difference (t = 4.82, *p* < 0.005). These indicate that the ASFV-SC-T7 group can induce a robust innate immune response.

Moreover, previous studies have indicated that monocytes contribute to the activation of CD8^+^ T cells [30]. On day 3 post primary immunization, the proportion of CD8^+^ T cells in PBMCs was significantly higher in the ASFV-SC-T7 group (18.6 ± 1.72)% than in the control group (7.6 ± 0.85)% (t = 29.59, *p* < 0.025; Figure 3C). By day 14 post-primary immunization, the ASFV-SC group also exhibited a significant increase in CD8^+^ T cells (10.3 ± 0.79)% relative to controls (6.7 ± 1.59)% (t = 4.917, *p* < 0.025).

Unlike live viral vectors that facilitate endogenous antigen expression, T7 phage-displayed vaccines likely promote CD8^+^ T-cell activation through the cross-presentation of exogenous antigens. The observed response in the ASFV-SC subunit group may also be explained by partial antigen phagocytosis and subsequent cross presentation.

Due to considerable baseline variation in CD4^+^ T cell levels, with pre-immunization values of (15.0 ± 3.10)% in controls and (23.1 ± 3.76)% in the ASFV-SC-T7 group (t = 2.06, *p* < 0.005), focused analysis of CD4^+^ T cell responses was not feasible.

In summary, these results demonstrate that T7 VLP vaccines elicit strong humoral and cellular immunity. Although protection against genotype II ASFV was not achieved, these findings provide valuable insights for future ASFV vaccine design.

### 3.3. T7 Phage-Displayed ASFV Proteins Also Trigger Severe Viral Infection

**Figure 4:**
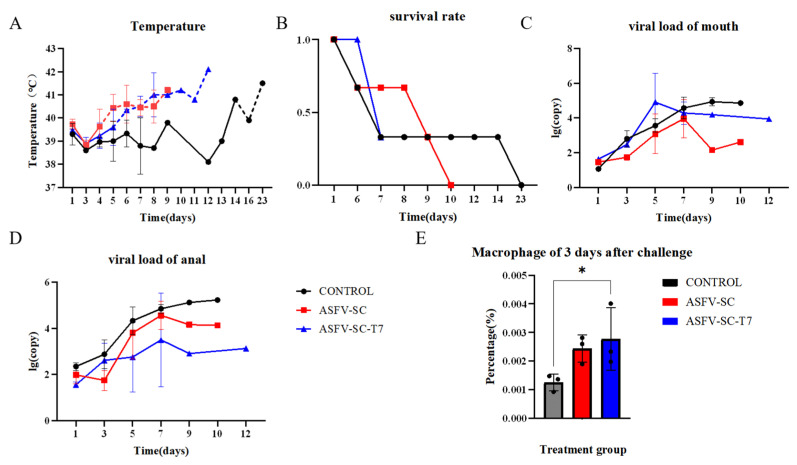
Effect of T7 phage-displayed ASFV candidate vaccine on pigs immunized with different vaccines. (**A**).The abscissa represents the time post-challenge (unit: day), and the ordinate denotes the rectal temperature (unit: °C). The dashed line indicates the upper limit of the normal body temperature in pigs (39.6 °C), with values exceeding this threshold defined as fever. Groups: Control group (black curve), ASFV-SC group (blue curve), ASFV-SC-T7 group (red curve). (**B**). Survival rate of experimental pigs following virus challenge with African swine fever virus (ASFV) strain 200HAD_50_ HuB/HH/2019. The abscissa represents the time post-challenge (unit: day), and the ordinate denotes the survival rate (where 0 indicates all deaths and 1 indicates all survivors). (**C**). Viral loading of swabs from the mouth of experimental pigs on days 0, 1, 3, 5, 7, or 9 following virus challenge. (**D**). Viral loading of swabs from the anus in experimental pigs on days 0, 1, 3, 5, 7, or 9 following virus challenge. (**E**) Proportion of macrophages in PBMCs on day 3 following the virus challenge. * *p* < 0.025.

Fourteen days post-booster immunization, all pigs were challenged intramuscularly with 200 HAD_50_ of the ASFV strain HuB/HH/2019. Oral and anal swabs were collected on days 1, 3, 5, 7, and 9 post-challenge as well as from moribund animals. The body temperature was monitored throughout the experimental period. Fever onset occurred on day 4 post challenge in the ASFV-SC group and on day 5 in the ASFV-SC-T7 group. In contrast, the control pigs did not exceed the normal temperature threshold (39.6 °C) until day 14 (Figure 4A). Despite the delayed fever response in the vaccinated groups, surface display of ASFV antigens on T7 phages did not confer effective protection. Survival analysis indicated that the control animals survived longer than those in the immunized group (Figure 4B).

Oral and anal swab collection from experimental pigs and viral load detection were performed on days 0, 1, 3, 5, 7, and 9 following virus challenge, and the results are shown in Figure 4C,D. Flow cytometry was used to detect differences among different groups of macrophages 3 days after challenge. On day 3 post-challenge, the proportion of macrophages in PBMCs was significantly higher in the ASFV-SC group (0.24 ± 2.93 × 10^−2^)% compared to controls (0.13 ± 4.79 × 10^−2^)% (t = 14.58, *p* < 0.025; two-tailed *t*-test; Figure 4E). This suggests that vaccination partially inhibits ASFV replication within macrophages, potentially triggering compensatory cellular proliferation.

**Figure 5:**
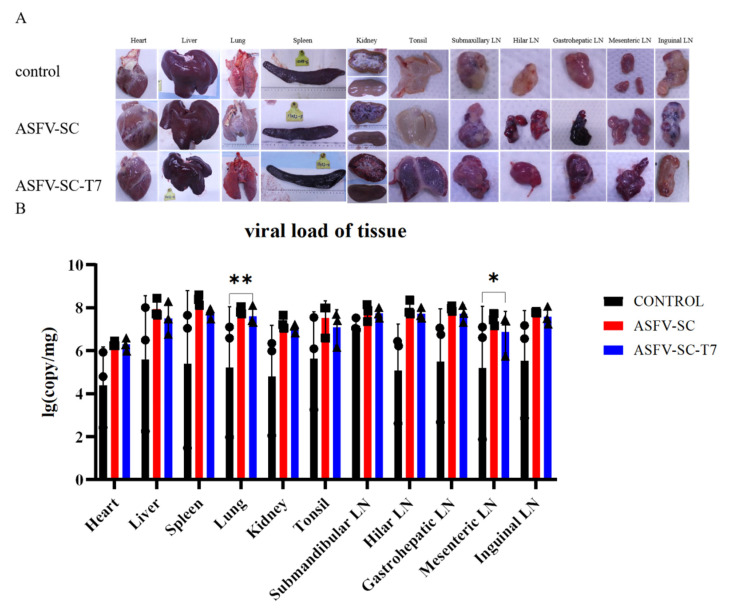
**Histopathology of pigs from different treatment groups at 7–8 days post-challenge.** (**A**). The organs displayed include the heart, spleen, kidney, liver, lung, tonsil, mandibular lymph node, hilar lymph node, hepatogastric lymph node, mesenteric lymph node, and inguinal lymph node. Each group (control group, ASFV-SC group, ASFV-SC-T7 group) corresponds to a column of gross anatomical images of the aforementioned organs. (**B**). Viral loading of tissues after autopsy. * *p* < 0.025, ** *p* < 0.005, n = 3.

Virulent ASFV infection causes extensive tissue damage and lesions in pigs [31]. Necropsy (Figure 5A) revealed typical ASF lesions across all groups, including severe congestion and multifocal diffuse hemorrhage in all sampled tissues. These lesions include hemorrhagic lymphadenitis, marble-like splenomegaly, myocardial petechiation, pulmonary consolidation, splenic hyperplasia with purple discoloration, and renal corticomedullary hemorrhage. Notably, lesion severity was more pronounced in the immunized groups. Both ASFV-SC and ASFV-SC-T7 groups exhibited more severe clinical manifestations than the control group (Figure 5A).

To assess ASFV replication across tissues, samples were collected from the heart, liver, spleen, lungs, kidneys, tonsils, and lymph nodes. Relative viral DNA contents across multiple tissues at necropsy in pigs following infection with ASFV were further measured by real-time PCR (Figure 5B); overall viral loads exceeded 1 × 10^4^ copies in all groups, indicating that immunization did not reduce the tissue viral burden in immunized groups compared to the controls. Specifically, the ASFV-SC-T7 group showed significantly higher viral loads in the lungs (Control: 5.2 ± 2.82 log_10_ copies; ASFV-SC-T7: 7.6 ± 0.44 log_10_ copies; *p* < 0.01, one-tailed *t*-test, t = 1.271) and mesenteric lymph nodes (Control: 5.2 ± 2.88 log_10_ copies/mg); ASFV-SC-T7: 6.8 ± 0.96 log_10_ copies/mg); *p* < 0.05, one-tailed *t*-test, t = 1.271).

## 4. Discussion

African swine fever virus (ASFV) is characterized by high pathogenicity, environmental stability, and transmission via both swine and ticks, leading to substantial global economic losses and highlighting the urgent need for effective vaccines [32]. Current vaccine approaches include inactivated vaccines, live-attenuated vaccines (LAVs), subunit vaccines, and nucleic acid-based formulations. Although traditional inactivated vaccines have failed to elicit protection, LAVs have shown efficacy against homologous strains; however, their utility is limited by poor cross-protection against heterologous strains and the risk of recombination [33]. Further development of LAVs is hampered by the absence of stable in vitro culture systems (e.g., monocyte/macrophage models) and labor-intensive screening required for targeted gene deletions [34]. Additionally, LAVs may cause chronic infections or revert to virulence [35], and multiple attenuated ASFV strains demonstrate a pronounced tendency for in vitro and in vivo recombination, giving rise to diverse and highly pathogenic chimeric viruses (chASFVs) [36].

Subunit vaccines that lack infectious materials offer a safer alternative to LAVs. They have demonstrated strong protection against various pathogens, including Brucella, influenza virus, and SARS-CoV-2 [37,38,39], and, more recently, have shown promise against monkeypox virus [40]. Given the structural similarities between ASFV and poxviruses, subunit vaccines represent a rational strategy to induce protective immunity against ASFV.

The choice of delivery vector for effective antigen presentation and immune activation is critical to subunit vaccine success. Phage-display technology has emerged as a robust platform for this purpose. T7 phage, in particular, has been widely employed as a vector for polypeptide/protein display because of its high stability, safety, strong immunogenicity, and tolerance to extreme physical and chemical conditions. The multivalent surface display system allows concurrent presentation of multiple antigenic epitopes, making it an attractive vehicle for polyvalent vaccine development [41].

ASFV p30 and p54 proteins play critical roles in mediating viral attachment to host cells. Simultaneous blockade of both proteins has a complementary effect on antibody-mediated protective immunity [42]. This aligns with reports showing that antibodies against p12 and p72 inhibit viral binding, whereas anti-p30 antibodies prevent viral internalization, suggesting that targeting multiple proteins can disrupt successive stages of the viral infection process, thereby improving protection [43].

The lack of protection observed in the ASFV-SC and ASFV-SC-T7 groups could be attributed to several factors. These include the use of a high-dose challenge with a virulent genotype II ASFV strain via the intramuscular route, a non-physiological model known to be highly stringent under which even commercially available live-attenuated vaccines (e.g., ASFV-G-ΔI177L and ASFV-G-ΔMGF in Vietnam) show limited efficacy. Therefore, the potential of T7 phage display technology in ASFV vaccine development should not be overlooked. Furthermore, the structural complexity of ASFV and the current lack of clearly defined protective antigens may have resulted in suboptimal antigen selection. Some studies suggest that antigens such as CD2v, P72, and P54 could potentially mediate antibody-dependent enhancement (ADE) of infection, worsening the disease in vaccinated animals [44], which might explain the severe clinical manifestations in the immunized groups. Thus, in addition to optimizing the immunization routes and regimens, rational antigen selection is essential. Given the structural similarities between ASFV and poxviruses, future efforts may benefit from leveraging antigen design strategies developed for poxvirus vaccines [45].

Although the limited protection observed here underscores the challenges in ASFV vaccinology, including stringent challenge models, antigen selection, and ADE risks, it also provides a foundation for further investigation of T7 phage-based vaccine platforms and basic ASFV virology. Building on these results, this study extended its focus to the biological features of ASFV and the specific responses in the ASFV-SC-T7 group, with the aim of situating these findings within the broader context of ASFV infection and control.

This study provides a comprehensive investigation of ASFV, a major threat to the global swine industry. The virus primarily targets the porcine mononuclear phagocyte system and its complex invasion mechanisms complicate disease control. The ASFV-SC-T7 group, the central focus of this study, elicited several promising immune responses during the trial.

Notably, this group induces strong humoral immunity, a key defense mechanism through which antibodies can block viral entry and spread. Consistent with previous reports, ASFV proteins, including P72, CD2v, P12, P54, D117L, and P30, are known to elicit neutralizing antibodies that inhibit viral adsorption, internalization, and release [46]. The robust antibody response triggered by the ASFV-SC-T7 formulation likely confers protection via similar mechanisms.

In terms of cellular immunity, the ASFV-SC-T7 group effectively promoted T-cell activation and proliferation. T cell-mediated responses play a critical role in defense against ASFV infection. Flow cytometry analysis revealed significantly elevated CD8^+^ T cell counts in the vaccinated group compared to the controls at multiple time points [47]. Previous studies have established a strong correlation between antigen-specific CD8^+^ T cell expansion and protection against lethal ASFV challenge, underscoring the importance of cytotoxic T cells in antiviral immunity [48]. The marked increase in CD8^+^ T cells observed here indicates that the ASFV-SC-T7 formulation enhances the host cellular immune defense against ASFV.

Macrophages are the primary target cells of ASFV and are critically involved in the severity of infection. Data from this study suggest that the ASFV-SC-T7 vaccine partially inhibits ASFV infection in macrophages. Since infection of these cells facilitates extensive viral replication and impairs innate immune function, partial containment of macrophage infection may help preserve immunological integrity and reinforce the host’s defense barrier.

In summary, the construction of African swine fever virus (ASFV) virus-like particles (VLPs) based on T7 phage display not only confirms the platform’s feasibility via distinct immunological advantages but also provides critical insights for addressing current limitations and guiding the optimization of ASFV vaccines.

This platform exhibits prominent immunogenic potential by mediating comprehensive immune activation and interfering with multiple ASFV infection stages, including inducing humoral immunity (e.g., antigen-specific antibody production), promoting cellular immunity (e.g., monocyte activation and CD8^+^ T cell proliferation, as validated herein), and partially inhibiting macrophage infection—the primary target cell of ASFV. Given the structural complexity (e.g., multiple antigenic proteins) and intricate pathogenic mechanisms of ASFV that have impeded licensed vaccine development, the observed immunogenic effects fully validate the T7 phage-derived VLP as a viable ASFV vaccine candidate platform.

Notably, the “incomplete protection” observed in this study is attributable to multiple non-platform-related factors. First, regarding the challenge model stringency, the 200 HAD_50_ intramuscular challenge dose exceeds the viral load in natural ASFV infections (intranasal/oral route, <10 HAD_50_); even potent gene-deleted live vaccines fail to achieve full protection under this high-dose model, suggesting the “non-protection” outcome may derive from the model rather than the platform itself [33,35]. Second, concerning antigen combination limitations, combined with Figure 5 data showing higher viral loads in the lungs and mesenteric lymph nodes of immunized groups, we hypothesize that the CD2v/P72 antigens may mediate antibody-dependent enhancement (ADE). This is supported by previous findings that anti-ASFV A137R antibodies can induce ADE [44]; confirmation of this hypothesis would explain the more severe pathological damage in immunized groups and underscores the necessity of subsequent ADE-risk antigen exclusion. Third, regarding immune memory, challenge at 14 days post-booster may be insufficient for establishing long-term memory immunity (e.g., memory B/T cell generation). Previous studies have demonstrated that SARS-CoV-2 nanoparticle vaccines require two boosters to induce robust memory responses [39], indicating optimization of immunization schedules (e.g., increased booster frequency) is critical for improving protective efficacy.

These findings provide important implications for ASFV vaccine development, supporting a “two-step strategy” based on this platform. Step 1 focuses on antigen optimization: leveraging the platform’s antigen display flexibility to screen ADE-free protective antigens (e.g., deleting CD2v and incorporating novel antigens such as E120R/H124R). Step 2 involves immunization regimen refinement: building on the platform’s verified capacity for monocyte activation and CD8^+^ T cell proliferation, optimize regimens via increased boosters or adjuvant combination to enhance long-term memory immunity. In conclusion, the T7 phage-displayed ASFV VLP platform holds significant promise for advancing ASFV vaccine development, with future research prioritizing the aforementioned limitations to fully exploit its potential.

## 5. Conclusions

This study constructed T7 phage virus-like particles (VLPs) displaying 5 key African swine fever virus (ASFV) proteins (P30, P54, P72, K145R, and CD2v), and systematically evaluated their immunogenicity and protective efficacy against virulent ASFV challenge in a swine model. The main conclusions are as follows:

T7 Phage VLPs Effectively Activate Humoral and Cellular Immune Responses in Pigs. ELISA results showed that compared with the group immunized with ASFV proteins alone (ASFV-SC group), the T7 phage antigen-display group (ASFV-SC-T7 group) induced more durable and high-titer antigen-specific antibodies (e.g., P30 and P72 antibodies) after booster immunization. Flow cytometry analysis confirmed that the ASFV-SC-T7 group significantly increased the proportion of monocytes in peripheral blood mononuclear cells (PBMCs) and promoted CD8^+^ T cell proliferation. These findings indicate that this platform can simultaneously activate innate immunity and adaptive cellular immunity.

T7 Phage VLPs Fail to Provide Protective Immunity Against Virulent ASFV. Although the ASFV-SC-T7 group showed a delayed onset of fever compared with the control group after challenge (day 5 vs. day 14), all pigs in the immunized groups developed typical ASF pathological lesions, such as splenomegaly with hemorrhage and hemorrhagic inflammation of lymph nodes. Additionally, the viral loads in the lungs and mesenteric lymph nodes of the immunized groups were significantly higher than those in the control group. These results suggest that this vaccine candidate cannot block the replication and pathogenicity of virulent ASFV.

The Study Provides Key References for ASFV Vaccine Development. This study confirms the feasibility of using T7 phages as a multi-antigen delivery platform for ASFV, and the potent immune response induced by this platform provides a basis for subsequent optimization. Meanwhile, it reveals that the current antigen combination (P30/P54/P72/K145R/CD2v) may carry the risk of “antibody-dependent enhancement (ADE)”, as evidenced by more severe pathological damage in the immunized groups. This indicates that future research should integrate the mechanisms of ASFV immune escape to screen for better antigen combinations (e.g., excluding antigens that may trigger ADE) and optimize immunization protocols (e.g., adjusting dosages or adding new adjuvants) to improve protective efficacy.

## Data Availability

Data are contained within the article.

## Supplementary Materials

The following supporting information can be downloaded at: https://www.mdpi.com/article/10.3390/vaccines14010004/s1.

## References

[1] Wang Y., Kang W., Yang W., Zhang J., Li D., Zheng H. (2021). Structure of African Swine Fever Virus and associated molecular mechanisms underlying infection and immunosuppression: A review. Front. Immunol..

[2] Zhou X., Li N., Luo Y., Liu Y., Miao F., Chen T., Zhang S., Cao P., Li X., Tian K. (2018). Emergence of African Swine Fever in China in 2018. Transbound. Emerg. Dis..

[3] Mighell E., Ward M.P. (2021). African Swine Fever spread across Asia, 2018–2019. Transbound. Emerg. Dis..

[4] Lv T., Xie X., Song N., Zhang S., Ding Y., Liu K., Diao L., Chen X., Jiang S., Li T. (2022). Expounding the role of ticks in African swine fever virus transmission and seeking effective prevention measures: A review. Front. Immunol..

[5] Xian Y., Xiao C. (2020). The Structure of ASFV Advances the Fight against the Disease. Trends Biochem. Sci..

[6] Wang N., Zhao D., Wang J. (2020). Novel insights into cryo-EM structure of African Swine Fever Virus. Sci. Found. China.

[7] Costard S., Wieland B., de Glanville W., Jori F., Rowlands R., Vosloo W., Roger F., Pfeiffer D.U., Dixon L.K. (2009). African swine fever: How can global spread be prevented?. Philos. Trans. R. Soc. Lond. B Biol. Sci..

[8] Andrés G., García-Escudero R., Viñuela E., Salas M.L., Rodríguez J.M. (2001). African swine fever virus structural protein pE120R is essential for virus transport from assembly sites to plasma membrane but not for infectivity. J. Virol..

[9] Neilan J.G., Zsak L., Lu Z., Burrage T.G., Kutish G.F., Rock D.L. (2004). Neutralizing antibodies to African swine fever virus proteins p30, p54, and p72 are not sufficient for antibody-mediated protection. Virology.

[10] Alejo A., Matamoros T., Guerra M., Andrés G. (2018). A Proteomic Atlas of the African Swine Fever Virus Particle. J. Virol..

[11] Chandana M.S., Nair S.S., Chaturvedi V.K., Abhishek Pal S., Charan M.S.S., Balaji S., Saini S., Vasavi K., Deepa P. (2024). Recent progress and major gaps in the vaccine development for African swine fever. Braz. J. Microbiol..

[12] Stone S.S., Hess W.R. (1967). Antibody response to inactivated preparations of African swine fever virus in pigs. Am. J. Vet. Res..

[13] Walczak M., Juszkiewicz M., Szymankiewicz K., Szczotka-Bochniarz A., Woźniakowski G. (2022). ASF -survivors’ Sera Do Not Inhibit African Swine Fever Virus Replication in Vitro. J. Vet. Res..

[14] Jancovich J.K., Chapman D., Hansen D.T., Robida M.D., Loskutov A., Craciunescu F., Borovkov A., Kibler K., Goatley L., King K. (2018). Immunization of Pigs by DNA Prime and Recombinant Vaccinia Virus Boost To Identify and Rank African Swine Fever Virus Immunogenic and Protective Proteins. J. Virol..

[15] Argilaguet J.M., Pérez-Martín E., Nofrarías M., Gallardo C., Accensi F., Lacasta A., Mora M., Ballester M., Galindo-Cardiel I., López-Soria S. (2012). DNA vaccination partially protects against African swine fever virus lethal challenge in the absence of antibodies. PLoS ONE.

[16] Sunwoo S.Y., Pérez-Núñez D., Morozov I., Sánchez E.G., Gaudreault N.N., Trujillo J.D., Mur L., Nogal M., Madden D., Urbaniak K. (2019). DNA-Protein Vaccination Strategy Does Not Protect from Challenge with African Swine Fever Virus Armenia 2007 Strain. Vaccines.

[17] Bosch-Camós L., López E., Rodriguez F. (2020). African swine fever vaccines: A promising work still in progress. Porc. Health Manag..

[18] Sereda A.D., Balyshev V.M., Kazakova A.S., Imatdinov A.R., Kolbasov D.V. (2020). Protective Properties of Attenuated Strains of African Swine Fever Virus Belonging to Seroimmunotypes I-VIII. Pathogens.

[19] Netherton C.L., Goatley L.C., Reis A.L., Portugal R., Nash R.H., Morgan S.B., Gault L., Nieto R., Norlin V., Gallardo C. (2019). Identification and Immunogenicity of African Swine Fever Virus Antigens. Front. Immunol..

[20] Reis A.L., Goatley L.C., Jabbar T., Lopez E., Rathakrishnan A., Dixon L.K. (2020). Deletion of the Gene for the Type I Interferon Inhibitor I329L from the Attenuated African Swine Fever Virus OURT88/3 Strain Reduces Protection Induced in Pigs. Vaccines.

[21] O’Donnell V., Holinka L.G., Sanford B., Krug P.W., Carlson J., Pacheco J.M., Reese B., Risatti G.R., Gladue D.P., Borca M.V. (2016). African swine fever virus Georgia isolate harboring deletions of 9GL and MGF360/505 genes is highly attenuated in swine but does not confer protection against parental virus challenge. Virus Res..

[22] Reis A.L., Goatley L.C., Jabbar T., Sanchez-Cordon P.J., Netherton C.L., Chapman D.A.G., Dixon L.K. (2017). Deletion of the African Swine Fever Virus Gene DP148R Does Not Reduce Virus Replication in Culture but Reduces Virus Virulence in Pigs and Induces High Levels of Protection against Challenge. J. Virol..

[23] Goatley L.C., Reis A.L., Portugal R., Goldswain H., Shimmon G.L., Hargreaves Z., Ho C.S., Montoya M., Sánchez-Cordón P.J., Taylor G. (2020). A Pool of Eight Virally Vectored African Swine Fever Antigens Protect Pigs Against Fatal Disease. Vaccines.

[24] Jacobs-Lorena M., Cha S.J. (2024). Unbiased phage display screening identifies hidden malaria vaccine targets. Emerg. Microbes Infect..

[25] Hess K.L., Jewell C.M. (2019). Phage display as a tool for vaccine and immunotherapy development. Bioeng. Transl. Med..

[26] Deng X., Wang L., You X., Dai P., Zeng Y. (2018). Advances in the T7 phage display system (Review). Mol. Med. Rep..

[27] Wong C.L., Sieo C.C., Tan W.S. (2013). Display of the VP1 epitope of foot-and-mouth disease virus on bacteriophage T7 and its application in diagnosis. J. Virol. Methods.

[28] Zuo X., Peng G., Xia Y., Xu L., Zhao Q., Zhu Y., Wang C., Liu Y., Zhao J., Wang H. (2023). A quadruple fluorescence quantitative PCR method for the identification of wild strains of african swine fever and gene-deficient strains. Virol. J..

[29] Murphy D.M., Cox D.J., Connolly S.A., Breen E.P., Brugman A.A., Phelan J.J., Keane J., Basdeo S.A. (2023). Trained immunity is induced in humans after immunization with an adenoviral vector COVID-19 vaccine. J. Clin. Investig..

[30] Li Y., Sun R., Li S., Tan Z., Li Z., Liu Y., Guo Y., Huang J. (2023). ASFV proteins presented at the surface of T7 phages induce strong antibody responses in mice. J. Virol. Methods.

[31] Elewaut A., Estivill G., Bayerl F., Castillon L., Novatchkova M., Pottendorfer E., Hoffmann-Haas L., Schönlein M., Nguyen T.V., Lauss M. (2025). Cancer cells impair monocyte-mediated T cell stimulation to evade immunity. Nature.

[32] Zhao D., Liu R., Zhang X., Li F., Wang J., Zhang J., Liu X., Wang L., Zhang J., Wu X. (2019). Replication and virulence in pigs of the first African swine fever virus isolated in China. Emerg. Microbes Infect..

[33] OIE African Swine Fever—World Organization for Animal Health. WOAH—World Organisation for Animal Health. https://www.woah.org/en/disease/african-swine-fever.

[34] Diep N.V., Duc N.V., Ngoc N.T., Dang V.X., Tiep T.N., Nguyen V.D., Than T.T., Maydaniuk D., Goonewardene K., Ambagala A. (2024). Genotype II Live-Attenuated ASFV Vaccine Strains Unable to Completely Protect Pigs against the Emerging Recombinant ASFV Genotype I/II Strain in Vietnam. Vaccines.

[35] Fuchs W., Assad-Garcia N., Abkallo H.M., Xue Y., Oldfield L.M., Fedorova N., Hübner A., Kabuuka T., Pannhorst K., Höper D. (2025). A synthetic genomics-based African swine fever virus engineering platform. Sci. Adv..

[36] van den Born E., Olasz F., Mészáros I., Göltl E., Oláh B., Joshi J., van Kilsdonk E., Segers R., Zádori Z. (2025). African swine fever virus vaccine strain Asfv-G-∆I177l reverts to virulence and negatively affects reproductive performance. NPJ Vaccines.

[37] Kitamura T., Masujin K., Ikezawa M., Ambagala A., Kokuho T. (2025). Generation of Chimeric African Swine Fever Viruses Through In Vitro and In Vivo Intergenotypic Gene Complementation. Vaccines.

[38] Gupta S., Singh D., Gupta M., Bhatnagar R. (2019). A combined subunit vaccine comprising BP26, Omp25 and L7/L12 against brucellosis. Pathog. Dis..

[39] Kompier R., Neels P., Beyer W., Hardman T., Lioznov D., Kharit S., Kostinov M. (2022). Analysis of the safety and immunogenicity profile of an azoximer bromide polymer-adjuvanted subunit influenza vaccine. F1000Research.

[40] He C., Yang J., Hong W., Chen Z., Peng D., Lei H., Alu A., He X., Bi Z., Jiang X. (2022). A self-assembled trimeric protein vaccine induces protective immunity against Omicron variant. Nat. Commun..

[41] Wen Y., Deng S., Wang T., Gao M., Nan W., Tang F., Xue Q., Ju Y., Dai J., Wei Y. (2024). Novel strategy for Poxviridae prevention: Thermostable combined subunit vaccine patch with intense immune response. Antivir. Res..

[42] Kumarasamy J., Ghorui S.K., Gholve C., Jain B., Dhekale Y., Gupta G.D., Damle A., Banerjee S., Rajan M.G.R., Kulkarni S. (2021). Production, characterization and in-vitro applications of single-domain antibody against thyroglobulin selected from novel T7 phage display library. J. Immunol. Methods.

[43] Gómez-Puertas P., Rodríguez F., Oviedo J.M., Brun A., Alonso C., Escribano J.M. (1998). The African swine fever virus proteins p54 and p30 are involved in two distinct steps of virus attachment and both contribute to the antibody-mediated protective immune response. Virology.

[44] Ruiz-Gonzalvo F., Rodríguez F., Escribano J.M. (1996). Functional and immunological properties of the baculovirus-expressed hemagglutinin of African swine fever virus. Virology.

[45] Zhai H., Gao Y., Zhu Y., Hou Q., Wan N., Wang T., Li S., Zhao D., Qiu H.-J., Li Y. (2025). Anti-pA137R antibodies exacerbate the pathogenicity of African swine fever virus in pigs. J. Virol..

[46] Yuan F., Cui J., Wang T., Qin J., Jeon J.H., Ding H., Whittaker C.A., Xu R., Cao H., Chen J. (2025). Selection, Design, and Immunogenicity Studies of ASFV Antigens for Subunit mRNA Cocktail Vaccines with Specific Immune Response Profiles. ACS Infect. Dis..

[47] Wang G., Xie M., Wu W., Chen Z. (2021). Structures and Functional Diversities of ASFV Proteins. Viruses.

[48] Crozat K., Tamoutounour S., Vu Manh T.P., Fossum E., Luche H., Ardouin L., Guilliams M., Azukizawa H., Bogen B., Malissen B. (2011). Cutting edge: Expression of XCR1 defines mouse lymphoid-tissue resident and migratory dendritic cells of the CD8α+ type. J. Immunol..

